# Revisiting the immune landscape post spinal cord injury: More than black and white

**DOI:** 10.3389/fnagi.2022.963539

**Published:** 2022-12-07

**Authors:** Liu Zhen-Gang, Yang Fan, Shi Jingwei, Chang Pengyu, Yu Shengman, Zhang Bo-Yin

**Affiliations:** ^1^Department of Orthopaedics, China-Japan Union Hospital of Jilin University, Changchun, China; ^2^Department of Laboratory Medicine Center, China-Japan Union Hospital of Jilin University, Changchun, China; ^3^Radiotherapy Department, The First Bethune Hospital of Jilin University, Changchun, China; ^4^School of Laboratory Medicine, Beihua University, Jilin, China

**Keywords:** spinal cord injury, central nervous system, immune cells, glial cells, immunotherapy

## Abstract

Spinal cord injury (SCI) induced catastrophic neurological disability is currently incurable, especially in elderly patients. Due to the limited axon regeneration capacity and hostile microenvironment in the lesion site, essential neural network reconstruction remains challenging. Owing to the blood–spinal cord barrier (BSCB) created immune cells and cytokines isolation, the immune elements were incorrectly recognized as innocent bystanders during the SCI pathological process traditionally. Emerging evidence demonstrated that the central nervous system (CNS) is an “immunological quiescent” rather than “immune privileged” area, and the CNS-associated immune response played mixed roles which dedicate beneficial and detrimental contributions throughout the SCI process. Consequently, coordinating double-edged immunomodulation is vital to promote tissue repair and neurological recovery post-SCI. The comprehensive exploration and understanding of the immune landscape post-SCI are essential in establishing new avenues for further basic and clinical studies. In this context, this review summarizes the recent significant breakthroughs in key aspects of SCI-related immunomodulation, including innate and adaptive immune response, immune organ changes, and holistic immune status modification. Moreover, the currently existing immune-oriented therapies for SCI will be outlined.

## Introduction

Spinal cord injury (SCI) is a catastrophic clinical syndrome that results in permanent neurological disability and fatal complications, especially among elderly patients. The SCI occurrence rate has increased in recent years as an adverse effect of economic and industrial development. In 2016, the estimated global prevalence rate of SCI was 368 cases per 100,000 population, and the primary causes included falls and traffic injuries (Feigin et al., 2019). The annual SCI medical expenditure in the United States was up to US $1.7 billion. Notably, the indirect costs of each patient were more than US $70,000 (Lo et al., 2021). Unfortunately, SCI-triggered devastating neurological impairment currently has no cure.

Pathophysiologically, the poor functional restoration of SCI can be generally attributed to various internal and external elements (Zhang B. Y. et al., 2020). Internally, the restrained capacity of the adult axon regeneration and sheath remyelination after SCI hamper the neural circuit reconnection. Externally, the glial activation and immune response onset make the microenvironment intractable, thereby suppressing tissue repair and axon regrowth (Bo-Yin et al., 2021). Neurological recovery demands the valid organization of internal and external factors. Previous studies on axon regeneration or inhibitory glial reaction have identified a spectrum of potential regeneration-related candidates (Liu et al., 2021). However, an efficacious strategy to rescue neurological disability remains to be developed.

Among these elements, the roles that immune regulation plays in the SCI repair process are somewhat blind spots that have attracted massive attention. Traditionally, the central nervous system (CNS), including the spinal cord, was recognized as an “immune-privileged area” owing to the blood–spinal cord barrier (BSCB) created by immune cells and cytokines isolation. However, recent studies have demonstrated that the CNS is an “immunologically quiescent” area rather than “immune privileged,” as it is equipped with quiescent immune cells capable of triggering comprehensive immune responses when required (Louveau et al., 2015). Under the SCI scenario, the quiescent immune system is provoked spontaneously, and multiple immune responses can be activated and amplified *via* various mechanisms (Al Mamun et al., 2021). During this process, various immune cell types, including innate glial cells, peripheral immune cells, and lymphocytes, played versatile roles in the SCI repair process. First, the spinal cord resident glial cells and infiltrated immune cells innately exert immediate immunomodulation after SCI, like the innate immune response against pathogens, which directly maneuvers the inflammation-associated cytokines and chemokines released in the early stage of SCI (Table 1). Moreover, the peripheral and infiltrated lymphocytes modify the adaptive immune response and play essential roles in neurological function repair. Additionally, as the development and storage sites of immune cells, immune organs are indispensable for immune function maintenance. SCI-induced immune reactions in immune organs, such as the spleen and gut, alter the holistic immune response status, especially in the SCI chronic stage. Consequently, by orchestrating the SCI-related critical pathophysiological changes, such as inflammatory infiltration, glial response, and axon regeneration, SCI-related immune response dedicates beneficial and detrimental contributions depending on the specific spatiotemporal environment. Although the regulatory mechanism is still not fully understood, the way to coordinate the double-edged immunomodulation is of great importance to post-SCI tissue repair and neurological recovery.

**Table 1:** SCI immunomodulation-associated cell types and roles.

	**Cell types**	**Cellular properties**		**Typical immune roles in CNS**
Innate immune	Astrocytes (AS)	Typical glial roles: Typical phenotypes:Secretion:	Trophic support and scar formationA1 neurotoxic typeA2 neuroprotective typeTrophic factors, cytokines, CSPG, GFAP	• AS Mediate the T cells phenotypes transfer via a cytokines dependent manner (eg. IL-1β, IL-6, IL-10, MCP-1), and perform the neuroprotective or neuroinflammaroty effect (Liu et al., 2019). • IL-15 upregulation in astrocytes exacerbate the CD8+ T and NK cells induced cerebral damage (Li et al., 2017). • AS compose the adventitial cuffs (AC) immune barrier structure, and mediate the immune cells infiltration and inflamatory response (Horng et al., 2017).
	Microglia (Mg)	Typical glial roles:Typical phenotypes:Secretion:Special types:	Phagocytosis and clearenceM1 proinflammatoryM2 anti-inflammatoryCytokines, complement proteinsTAMs	• TIM-3 is a critical immue regulator expressed by Mg (Koh et al., 2015). • Inhibiting the Mg expressed TIM-3 is neruoprotective (Koh et al., 2015). • M2 type TAMs produced TGF-β repress the CD8+ T cells and activate the Treg cells /MDSCs (Hambardzumyan et al., 2016).•Mg expressed C3 and C3R are related to the synaptic refinment and nerve circuit plasity, (The microglia synapses phagocytosis effect) (Hong et al., 2016). • Mg regultes AS phenotype transfering via expressing IL-6, TNF-α and Drp-1 (Joshi et al., 2019).
	Oligodendrocytes (OLs)	Typical glial roles:Secretion:	Myelination and trophic supportOMgp, MBP, MAG, MOG, cytokines	• OLs Mediate the NK cells and Th cells via HLA-E, and the immune response will induce the OLs survival and the demyelination (Zaguia et al., 2013). • The oligodendrocyte precursor cells (OPCs) is also antigen presenting cells (APC) cells in CNS (Kirby et al., 2019). • The IFN-γ can be presented to CD8+ T cells and induced the cytotoxicity reaction of CD8+ T cells toward OPC (Kirby et al., 2019). • OLs produced CD200 inhibits the Mg activation induced CNS neuroinflammation (Koning et al., 2009). OMgp and complement inaction modulate the myelin clearance and inflammatory progress (Brennan et al., 2015).
	Neutrophils	Typical roles:Secretion:Special types:	Phagocytosis and surveillanceCytokines, enzymes, MMPs, chemokinesCD14+Ly6G low neutrophils	• BLT1 deletion inhibits the neutrophils infiltration post SCI and thus promotes functional recovery (Saiwai et al., 2010). • The novel neutrophils subset (eg. CD14+Ly6G low) is neuroprotective (Sas et al., 2020). • SLPI The novel Neutrophils produced in the SCI early stage is neuroprotective and promote the SCI recovery (Tang et al., 2020).
	Monocytes derived macrophages (MDM)	Typical roles:Typical phenotypes:Secretion:	Phagocytosis and clearenceM1 proinflammatoryM2 anti-inflammatoryCytokines, endosomes, MMPs	• MDM produced IL-10 and IL-11 are benefical for the neurological functional recovery, and limits the neuroinflammation in lesion (Shechter et al., 2009). • MDM produced endosomes (eg. NOX2) promote the axon regenertaion by inhibiting the PTEN signaling pathway (Hervera et al., 2018). • MDM modulates the MMP-9 epression induce the ECM formation and epicenter tissue repair (Hong et al., 2017). • MDM and innate microglia coculture decrease the IL-1β expression, which promotes lesion site healing and the functional recovery (Greenhalgh et al., 2018).
Adaptive immune	B cells	Spinal resident group:Typical phenotypes:Secretion:	Meningeal lymphatic system pro-B, pre-B, immatureB, matureB, plasma cellsCytokines, antibodys, Immunoglobulin	• Glycoengineered anti-muCD20 mediated B cells blockade is conductve to SCI recovery (Casili et al., 2016). • The B cells knockout improved locomotor recovery post SCI (Jones, 2014; Casili et al., 2016). • The plasma cells mature deficiency and antibodies production impediment are neuroprotective (Jones, 2014).
	T cells	Spinal resident group:Typical phenotypes:Secretion:	Meningeal lymphatic systemCytotoxic T cells (CD8+), helper T cells (CD4+), regulatory T cells (FoxP3+)Cytokines	• In SCI, Th1 cells expressed TNF-α and IL-6 were upregulated and promote the M1 macrophages formation and activation, and thus deteriorate the lesion damage (Hirai et al., 2013). • Trauma induced autoimmunity promote the MBP-T cells transfer into Th2 subtype which are beneficial for SCI (Hu et al., 2016). • Treg cells expressed IL-10 inhibits the M1 microglia transformation (Liesz et al., 2013). • Treg cells mediated neuroprotection is subject to the constraints of time and space (Raposo et al., 2014).

Thus, this review summarizes the recent significant progress in CNS immunomodulation studies. It describes the SCI-associated immune cells and organ-related responses and summarizes the currently existing immune-oriented therapies for treating SCI.

## The immune roles of spinal cord glial cells—Far beyond the glia

### The immune roles of innate glial cells in the spinal cord

**Figure 1:**
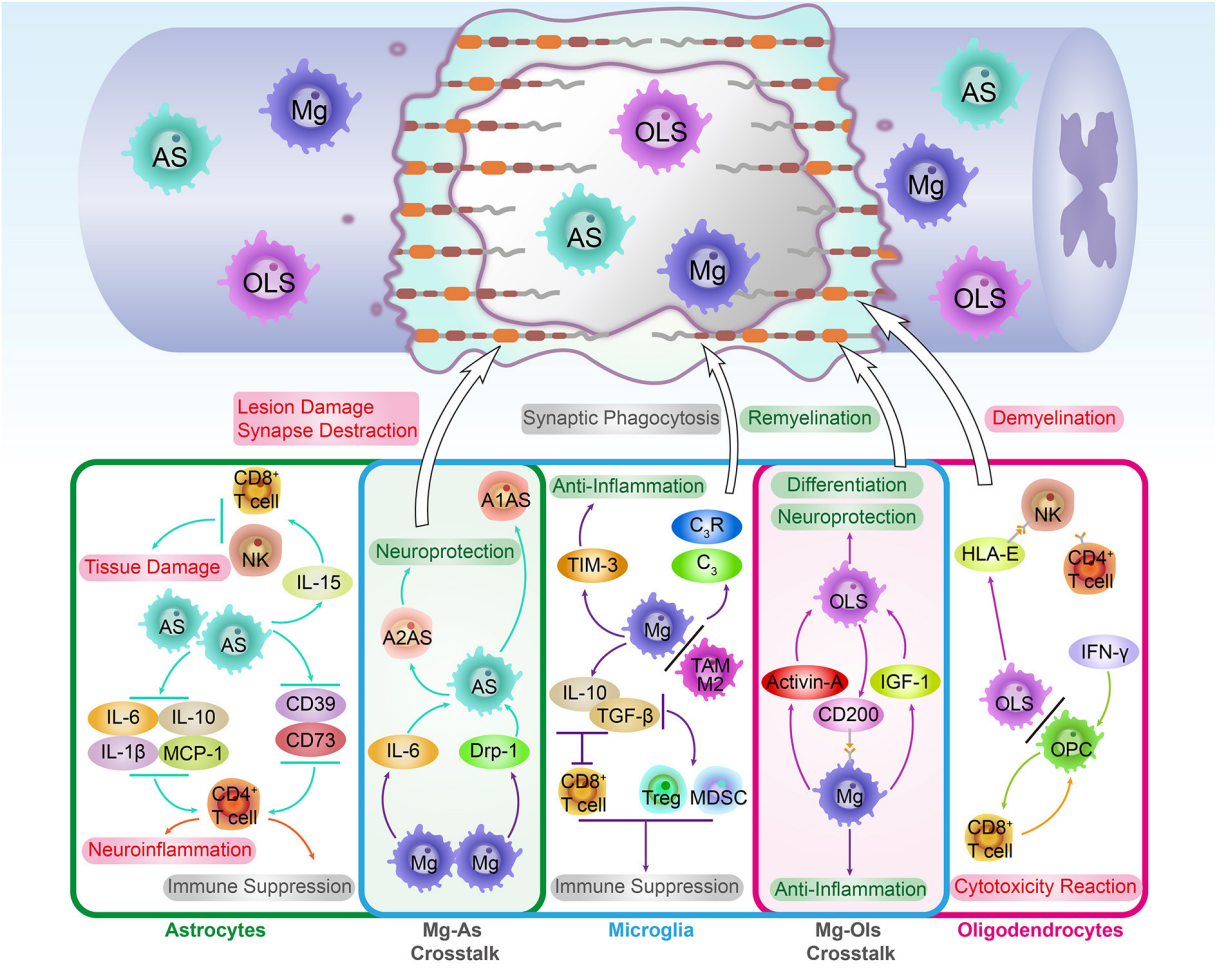
The immune roles and cross-talk of innate glial cells in the spinal cord. As the co-founder of CNS, the spinal cord's innate glial cells, including astrocytes, microglia, and oligodendrocytes, are essential for immune landscape establishment during SCI pathological repair process. From the SCI recovery point of view, these glial-related immune responses played mixed roles in the key steps of SCI pathological repair. To illustrate, by expressing pro-inflammatory and anti-inflammatory cytokines, glial cells modulate lymphocyte-induced neuroimmune functions. These functions could be beneficial and neuroprotective, which promote remyelination, axon regeneration, and glial differentiation. Instead, they also could be detrimental to SCI recovery, which aggravates tissue damage, cytotoxicity reaction, and demyelination. In addition, glial cells are still able to orchestrate the complement system activation and lymphocyte phenotypes transformation. Remarkably, the crosstalk among innate glial cells not only strengthens the communication between the CNS and immune system but supports the exploration of novel targets for SCI treatment. An important example of crosstalk is microglia-mediated astrocyte phenotype transformation, which induces the neuroprotective A2 phenotype astrocyte formation.

The spinal cord is primarily composed of two categories of cells: glial cells and neurons. Glial cells, including astrocytes, microglia, and oligodendrocytes, account for 90% of the total intraspinal cellular amount. The well-known functions of glial cells include nerve conduction activity maintenance and neuronal survival protection. Findings of recent studies indicate that glial cells also act as resident immune cells and play critical roles in the CNS's innate immune responses, especially in the spinal cord (Xu et al., 2020). Glial cells tend to exert immunomodulation innately, analogous to the innate immune response against pathogens. To illustrate, they directly regulate the inflammatory-related cytokine and chemokine expression without immune memory (besides microglia cells) and modulate the subsequent inflammatory cell infiltration and secondary injury inflammation cascade amplification (Figure 1).

#### The neuroimmune roles of astrocytes

Naïve astrocytes were found to transform into the reaction pool and scar pool in response to SCI. Shortly after SCI, the reactive astrocytes provided trophic factors and elements, which are beneficial for synapse formation and axon regeneration. Afterward, the reactive astrocytes formed glial scars, restraining the lesion site's inflammation during the earlier stage of the SCI repair process. However, with the accumulation of mature glial scars, the spinal lesion microenvironment irreversibly turned hostile, which inhibited axon regrowth (Zhang B. Y. et al., 2020). Astrocytes produced soluble inflammatory mediators, which were found to modulate immune cell recruitment and activation as neurotrophic cells. Therefore, besides the glial functions, astrocytes play considerable roles in CNS immune regulation.

Recent studies have demonstrated that astrocytes can mediate the T cell's polarization after CNS injury. The miR-409-3p and miR-1869 in astrocytes promote cytokine expression, such as IL-1β, IL-6, IL-10, and MCP-1, *via* the SOCS3/STAT3 pathway. Under the CNS injury condition, the astrocyte-oriented cytokine enrichment in the lesion induces the astrocyte-associated CD4^+^ T cells migration and polarization, which further results in neuroinflammation aggravation (Liu et al., 2019). Moreover, astrocytes also promote the plasma membrane ectonucleotidase expression (e.g., CD39, CD73) in activated CD4+ T cells, which promotes the T cells polarization to a Th17-like immunosuppressive phenotype during the neuroinflammation progression (Filipello et al., 2016). Additionally, under the CNS ischemic condition, astrocytes produced pro-inflammatory cytokine IL-15 up-regulation in astrocytes, which exacerbated the CD8+ T cells, and NK cells induced the immune-related cerebral damage. Instead, the deletion of astrocyte-derived IL-15 or IL-15 receptor knockdown alleviated the size of the lesion site and neurological dysfunction (Li et al., 2017).

In the CNS, the adventitial cuff (AC) structure is the perivascular terminal structure that hosts several kinds of immune cells. The integrity of AC expends the immune cell infiltration interfaces of immune organs when suffering from diseases such as infection and allergy (Dahlgren and Molofsky, 2019). Astrocytes around AC borders were demonstrated to serve as immune barriers against CNS inflammation invasion. The local astrocyte aggregation induced tight junction protein CLDN1 and CLDN4 upregulation, which encircled immune cells into AC borders when inflammatory infiltration occurred. Moreover, Sam et al. found that *in vivo* astrocyte-specific CLDN4 inactivation disrupted the astrocyte-mediated immune cell infiltration and deteriorated the lesion damage in a multiple sclerosis (MS) model (Horng et al., 2017).

#### The neuroimmune roles of microglia

Microglia are the first activated inflammatory responder in the lesion site after SCI. As the innate phagocytes in the CNS, microglia eliminate cellular debris and intracellular protein particles that may hinder remyelination. In addition, microglia also produce pro-inflammatory factors and neurotrophic factors that regulate the neuroinflammatory reaction. The SCI stress triggers the release of damage-associated molecular patterns (DAMPs) in the cytoplasm around the lesion site. Afterward, the DAMP signaling was recognized and captured by the pattern recognition receptors (PRRs) on microglia, and the DAMP/PRRs complex formed simultaneously. As a result, the complex stimulated microglia proliferation and triggered the microglia into the activated condition (Kigerl et al., 2014).

Activated microglia can be classified into two phenotypes, the immune-activated type (M1, pro-inflammatory) and the protective type (M2, anti-inflammatory). By releasing cytokines and inflammatory factors, the M1-type microglia trigger a neuroinflammatory cascade that turns the injury site into a hostile milieu that represses axon regeneration and neural survival. The SCI secondary injury turns microglia into the M1 type, which exaggerates the inflammatory response in the lesion site (Zhang B. Y. et al., 2020). In contrast, the branching morphology is the hallmark of the M2-type microglia, and the M2 microglia play beneficial roles after SCI by means of elevated phagocytic activity and anti-inflammatory cytokines expression (Bellver-Landete et al., 2019). To illustrate, by moderating the immune response of resident microglia, neuregulin-1 (Nrg-1) plays a neuroprotective role in SCI recovery. The systemic administration of Nrg-1 after SCI promotes the M2 phenotype formation at the SCI acute stage and increases the M1 phenotype formation during the SCI chronic stage (Alizadeh et al., 2018). Thus, the innate immune function of microglia after SCI was determined by the balance between the two phenotypes. In the CNS malignant tumor lesion, the microglia are involved in the tumor-associated microglia and macrophages (TAMs), which regulate tumor growth and immune response. Similar to CNS naive microglia, the M2 TAMs subtype cells have pro-tumorigenic and anti-inflammatory functions (Andersen et al., 2021). By secreting anti-inflammatory cytokines, such as TGF-β and IL-10, the M2-type TAM cells repressed the cytotoxic T cells and activated the regulatory T (Treg) cells and myeloid-derived suppressor cells. Consequently, the CNS tumor microenvironment turned into an immunosuppressive status that facilitated tumor growth and progression (Hambardzumyan et al., 2016).

Besides the innate immune response function and inflammatory roles, microglia also proved to possess immune memory in CNS inflammatory events. After experiencing the priming challenge of ligands, such as lipopolysaccharide (LPS) and β-glucan, microglia can be induced into immune training or immune tolerance status. Therefore, the immune response of primed microglia toward secondary stimulation could be activated or suppressed (Zhang et al., 2022). Yang et al. (2017) demonstrated that the distinct preconditioning protocols result in microglia's immune memory-related response, and the results provide promising treatment targets for microglia-associated diseases. For instance, the 2-day interval challenge protocol resulted in microglia immune training. However, the 7-day interval challenge protocol reversed the immune training into the immune tolerance status (Heng et al., 2021). Mechanically, the immune memory phenomenon of microglia results from the epigenetic remodeling navigated functional phenotype transformation. For instance, the LPS-induced gene expression was found to correlate with the enrichment of the permissive epigenetic markers in promoter regions, such as H3K4me3 and H3K27Ac (Zhang et al., 2022).

In addition to the inflammatory factors and cytokine secretion, microglia are competent in expressing special neuroimmune components. T-cell immunoglobulin and mucin domain protein-3 (Tim-3) are critical microglia-derived immune checkpoints closely related to CNS tissue damage and inflammation. It can serve as a therapeutic target for CNS disorders. In the brain, pathogens such as infectious disease or hypoxia–ischemia induced the Tim-3 up-regulation, which was recognized as an adverse factor in neurological recovery (Koh et al., 2015). A study found that the phagocytosis and secretion of microglia were modulated by Tim-3 activity. More particularly, Tim-3 activation promoted the microglia expression of inflammatory cytokines, such as TNF-α, IL-1β, and TGF-β. On the contrary, the Tim-3 blockade decreased the phagocytic activity of microglia (Wang et al., 2015). In addition, the Tim-3 blockade significantly reduced inflammatory cell infiltration and lesion volume in the cerebral hypoxia–ischemia model and improved neurological function (Koh et al., 2015). Furthermore, microglia still interact with complement proteins to regulate CNS function *via* immunological strategies (Cowan and Petri, 2018). For instance, microglia regulate synaptic refinement and nerve circuit projection by modulating the complement system-mediated synapse phagocytic effect. The effect was revealed to play a critical role in CNS diseases such as Alzheimer's disease and cerebral hypoperfusion (Zhang L. Y. et al., 2020). In this regard, the redundant synaptic cytoskeleton can be eliminated by microglia-expressed complement protein 3 (C3) and its receptor C3R (Hong et al., 2016).

#### The neuroimmune roles of oligodendrocytes

Oligodendrocytes are the CNS myelinating cells involved in both physiological and pathological processes. Previous studies primarily focused on the role of oligodendrocytes in myelination, and the immune function of oligodendrocytes is still underexplored (Dulamea, 2017).

Recently, the immunological profiles of oligodendrocytes were explored and recognized extensively. Fatma et al. reported that HLA-E, a subtype of non-classical MHC-1 factors, can be released by oligodendrocytes. By binding to the NKG2 receptor on NK cells and CD4^+^ T cells, HLA-E mediates the CNS immune responses toward oligodendrocytes. In the tissues of a patient with MS, the interaction between NKG2C^+^/CD4^+^ T cells and HLA-E^+^ oligodendrocytes induces the oligodendrocyte's death and demyelination modification (Zaguia et al., 2013). The differentiation of oligodendrocyte precursor cells (OPCs) rescues the oligodendrocyte loss following CNS injury or disease. Interestingly, OPCs also act as antigen-presenting cells (APCs) under neuroinflammatory conditions. When exposed to IFN-γ in the lesion, the MHC class-I presentation pathway of OPC was activated, and the antigen was cross-presented to CD8^+^ T cells. Therefore, the CD8^+^ T cell-mediated cytotoxicity reaction recognized OPCs as target cells and resulted in the OPC's sacrifice and extensive demyelination in the lesion site (Kirby et al., 2019).

### Crosstalk among glial cells

Recent studies discovered various types of crosstalk among different types of glial cells. The communication between the CNS and the immune system is strengthened *via* crosstalk. In addition, with the coordination of the glial cells, the phenotype transformation is carried out after nerve injury spontaneously, which modulates the immune response and inflammatory reaction in the lesion site and supports the establishment of novel discovery targets.

#### Microglia–astrocyte interaction

One of the well-known crosstalk is the microglia-mediated astrocyte phenotype transformation. The reactive astrogliosis and scar formation effect were recognized as typical characteristics of the neuroprotective reaction of the A2-type astrocytes. In CNS traumatic injury, the exposure of microglia-derived pro-inflammatory cytokines, such as IL-6 and TNF-α, promotes the A2 phenotype astrocyte transformation, which accelerates the protective astrogliosis in the lesion epicenter. In addition, microglia can still modulate the astrocyte-associated glial reaction and phenotype transformation by regulating the astrocyte-specific P2Y_1_ receptor expression (Shinozaki et al., 2017). After the CNS injury, the microglia are activated earlier than the astrocytes. Therefore, microglia-released mitochondria debris and neurotoxic proteins, such as dynamin-related protein1 (Drp-1), accumulated in the extracellular milieu. The enrichment of Drp-1 promoted the astrocytes transformed into A1 neurotoxic phenotype, which was characterized by pro-inflammatory factor secretion, synapse destruction, and lesion damage, and eventually directly propagated neuronal death in the CNS (Joshi et al., 2019).

#### Microglia–oligodendrocyte crosstalk

The bi-directional crosstalk between microglia and oligodendrocytes exist in both CNS development and pathological condition. On the one hand, microglia orchestrated the oligodendrocytes' differentiation, remyelination, and survival process. On the other hand, oligodendrocyte-expressed proteins regulated the microglial activity. Studies found that the microglia are necessary to regulate postnatal oligodendrocyte linage homeostasis and maturation, and the *in vivo* microglia depletion is detrimental to the quality and quantity of oligodendrocytes (Hagemeyer et al., 2017). In addition, activated microglia-secreted growth factors modulated the oligodendrocyte survival and lineage selection during the remyelination process. For instance, microglia produced IGF-1 exerted the neuronal protective function, and it rescued the cuprizone-induced oligodendrocytes apoptosis and promoted remyelination after CNS disease attack (Sierra et al., 2010).

Moreover, Miron et al. reported that polarized M2-type microglia secreted activin-A, promoting oligodendrocyte lineage differentiation and axon remyelination. In this context, the finding is probably one of the potential mechanisms of M2 phenotype microglia-induced neuroprotective properties during the CNS remyelination process (Miron et al., 2013).

Although microglia-derived factors, such as lipolytic enzymes, reactive oxygen, and excitotoxins, are necessary for pathogen clearance in the immune defense process, oligodendrocytes are highly sensitive to these factors. Oligodendrocytes in a hostile environment produce poor-quality myelin, which impedes the injured axon remyelination. Additionally, these microglia-released factors block OPC proliferation and induce OPC sacrifice (Peferoen et al., 2014).

CD200 is one of the membrane glycoproteins that contribute to the CNS immune suppressive milieu. Nathalie et al. found that the oligodendrocyte-expressed CD200 regulated the activity of microglia. By binding to its ligand CD200R located on the microglia surface, CD200 exerted the inhibitory regulation of microglia activity. On the contrary, the CD200 deletion resulted in the hyperactivity of microglia and CNS neuroinflammation (Koning et al., 2009).

#### Glial cell–myelin crosstalk

Myelin debris ubiquitously distributes in the lesion environment after SCI, which drives the ongoing neuroimmune activities. Microglia-mediated myelin debris clearance in the lesion environment facilitates axon regeneration and oligodendrocyte differentiation. As a receptor of damaged myelin lipid components, the TREM2 is expressed on the surface of microglia and is required for the myelin debris phagocytosis after SCI. A study reported that the mutations or deficiencies of TREM2 in the microglia inhibit the uptake and the subsequent phagocytosis of myelin fragments post-SCI (Poliani et al., 2015). Complement–myelin interaction was identified as another type of immune crosstalk post-SCI that contributed to the intraspinal inflammation regulation after injury. The binding of myelin fragments released oligodendrocyte myelin glycoprotein, and the complement initiated the complement activation and the complement-mediated opsonization. Once activated, complement C5a recruited the immune cells, such as macrophages and neutrophils, from the periphery blood to the injury site, which promoted myelin clearance and inflammatory process in the lesion (Brennan et al., 2015).

## The immune roles of peripheral immune cells—The unfavorable guys?

### BSCB disruption post-SCI

The blood–spinal cord barrier is a crucial barrier structure between the CNS and circulatory system, and it is composed of endotheliocytes, pericytes, astrocytic processes, intercellular connections, and membrane structures (Jin et al., 2021). Among these components, the endotheliocytes and the adherens junction proteins constitute a cellular barrier, which protects the spinal cord from harmful molecules. In addition, pericytes in spinal cord capillaries regulate the permeability of the BSCB by adjusting the blood flow status of the neurovascular system and modulating extracellular matrix (ECM) and growth factors. Previous studies demonstrated the sieve-like roles of the BSCB and considered it the key interface for molecular and neurotransmitter exchange of the spinal cord microenvironment (Cardoso et al., 2010). Of note, accumulating evidence suggests that the BSCB contributes to intraspinal immune homeostasis and CNS immune surveillance function.

**Figure 2:**
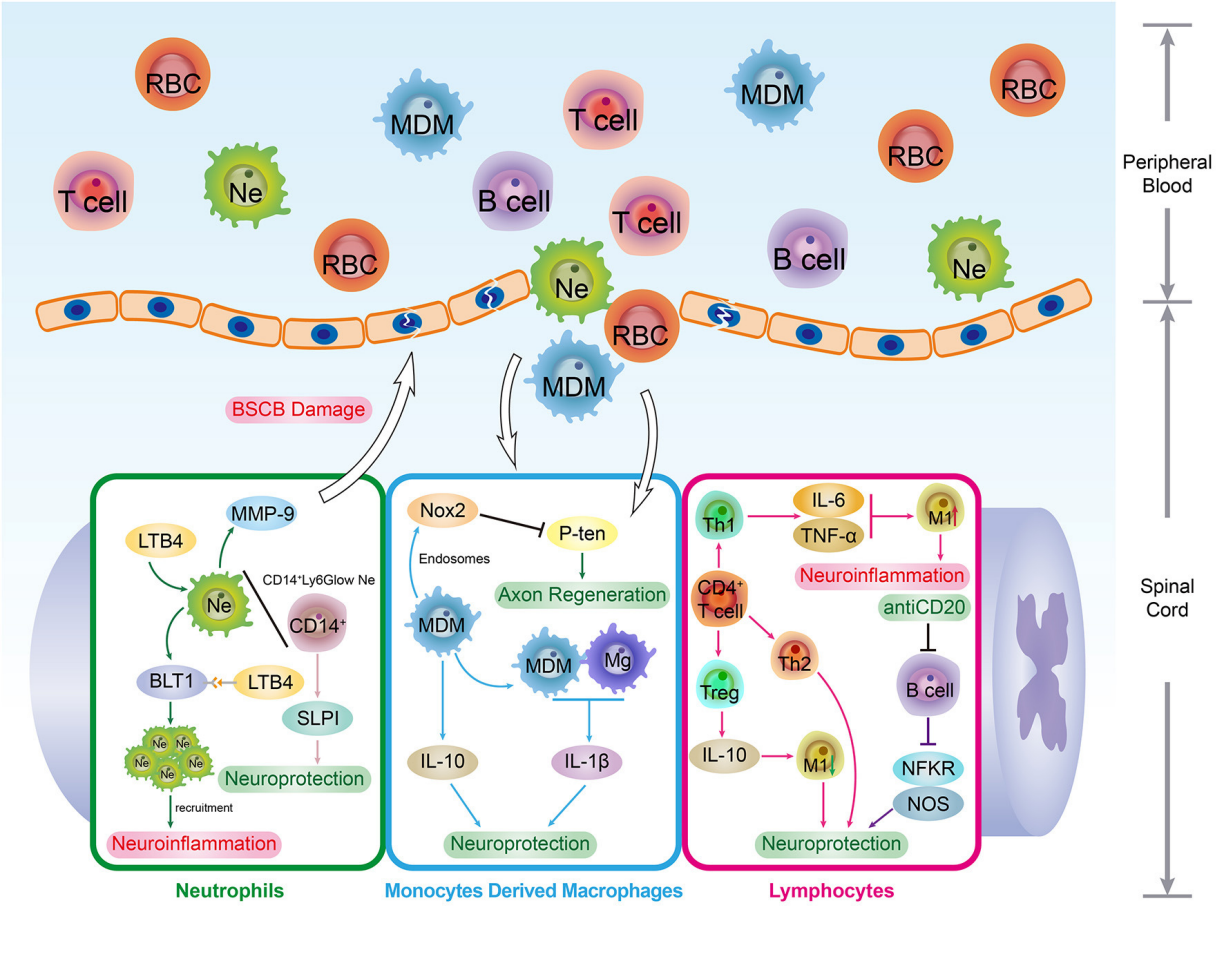
The immune roles of peripheral immune cells. The SCI events not only interrupt the BSCB integrity but break the intraspinal immune homeostasis. With the permeability elevation, peripheral immune cells such as neutrophils (Ne), monocytes derived macrophages (MDM) and lymphocytes are infiltrated in spinal cord lesions. In spinal cord lesions, these peripheral phagocytes. As the typical peripheral phagocytes, Ne and MDM cells also work as immune regulators in CNS. By interacting with immune factors in the spinal cord microenvironment, Ne and MDM played both positive and negative roles in regulating SCI recovery. In addition, the infiltration of lymphocytes enables the adaptive immune response to occur in the spinal cord, which provides significant immunomodulation for SCI pathological repair process. Interestingly, lymphocytes are competent in regulating the phenotype transformation of macrophages. To illustrate, the Th1 cells expressed cytokines such as TNF-α and IL-6 induce the transformation and activation of M1-type macrophages which results in extensive demyelination and neural tissue necrosis.

After an SCI attack, both the histological structure and immune homeostasis of the BSCB are affected dramatically. First, the irreversible histological structure disruption permits the exogenous immune cells to recruit and infiltrate into the lesion site through the compromised BSCB. For instance, peripheral inflammatory cell infiltration, such as neutrophils and macrophages, leads to neuroinflammatory and demyelination. Lymphocytic infiltration may trigger necrosis of neural tissue and activation of glial cells (Aubé et al., 2014). Moreover, SCI-induced “peripheral guys” infiltration disrupts the BSCB-built immune homeostasis microenvironment in the spinal cord. Under this circumstance, the intraspinal molecular and glycoprotein are liable to be recognized as exogenous antigens by infiltrated peripheral immune cells. Although these peripheral infiltrated cells were traditionally considered “unfavorable guys,” emerging evidence demonstrates that these peripheral partners play complex immune roles during the SCI injury recovery (Figure 2). Consequently, a comprehensive understanding of the peripheral infiltration-induced immune response and BSCB morphological changes post-SCI might be indispensable for developing novel medical strategies for SCI treatment.

### Peripheral neutrophils

Among these peripheral infiltrated cells, neutrophils are the first visitors that arrive at the spinal cord lesion within minutes post-injury (Wang, 2018). The typical role of the neutrophils is the phagocytic effect, which is essential for cellular and myelin debris clearance post-SCI (Zivkovic et al., 2021). Additionally, by secreting proteases, cytokines, and enzymes, neutrophils play versatile immune roles during the SCI. Studies found that inhibiting the recruitment of peripheral neutrophils and monocytes could not only reduce the oxidative stress associated with non-heme iron accumulation in the lesion but also downregulate the MMP-9 expression significantly. Under this circumstance, the long-term (28–42 days) neurological functional recovery after SCI improved markedly. In addition, a research group noted that the recruitment inhibition of neutrophils was conducive to protect the BSCB from MMP-9-induced disruption (Lee et al., 2011). Leukotriene B4 is one of the arachidonic acid metabolism productions. By interacting with neutrophils' leukotriene B4 receptor (BLT1), leukotriene B4 induces the recruitment of neutrophils. Studies found that the immunosuppressive effect of BLT1 deletion inhibited the post-injury neutrophil infiltration. Consequently, the inflammatory reaction and neuron apoptosis were limited to BLT1 deletion, thus promoting the recovery after SCI (Saiwai et al., 2010).

Although the neutrophil infiltration could exacerbate the SCI damage, recent evidence suggests that recruitment also exerts beneficial roles in the spinal cord lesion. Sas et al. discovered a novel subset of immature neutrophils, CD14^+^Ly6Glow, which possess neuroprotective and axon regenerative properties. The transplantation of the Ly6Glow promotes neuron survival and axon regeneration after CNS injury (Sas et al., 2020). The secretory leukocyte protease inhibitor (SLPI) is a neutrophil-produced serine protease inhibitor.

In the early stage of SCI, the SLPI's upregulation was detected in lesions, confirming the anti-inflammatory properties for neurological recovery (Zivkovic et al., 2021). In addition, by over-expressing the SLPI, Tang's group detected the neuroprotective role of SLPI post-SCI. These results suggest that the neutrophil-derived SLPI played a positive role in promoting the functional recovery of SCI (Tang et al., 2020).

### Peripheral monocyte-derived macrophages

As the most prevalent glial cells in the spinal cord, the macrophages in the lesion generally originate from two sources: the spinal cord resident microglia (the innate group) and infiltrated peripheral blood monocyte-derived macrophages (the exotic group, MDM). Peripheral blood monocytes were recruited to the spinal cord lesion spontaneously post-injury and peaked at 7 days post-injury. Afterward, the differentiated descendants of infiltrated monocytes, the exotic group MDM, started to accumulate and activate onsite. Studies found that the exotic group macrophage activation is of great importance to SCI recovery. For instance, by secreting anti-inflammatory cytokines, such as IL-10, infiltrated macrophages not only essentially promoted the neurological behavior performance post-SCI but also limited the inflammation-associated nerve damage (Shechter et al., 2009).

Recent studies demonstrated that macrophage-released endosomes, such as NOX2, can be recognized and transported retrogradely to soma post-DRG conditioning lesions. Then, the NOX2 enrichment inactivated the PTEN signaling pathway *via* intracellular oxidation, thereby stimulating axon regeneration (Hervera et al., 2018). Additionally, Hong et al. found that the macrophage-mediated ECM remodeling capacity was promoted by polymer hydrogel (imidazole-poly-organophosphazenes) microinjection. In a study, the exogenous hydrogel stimulated the macrophage's histamine receptor-mediated MMP-9 upregulation in the lesion site, which induced the fibronectin-rich ECM formation and bridged the physical barrier at the epicenter. Consequently, the post-traumatic cystic cavities were eliminated dramatically, and the axonal reinnervation and neurological function were largely improved (Hong et al., 2017). Interestingly, the interactions between the exotic and innate groups of macrophages also play beneficial roles post-SCI. The innate microglia-mediated pathological reactions post-SCI, such as phagocytic and inflammatory response, can be limited by exotic macrophage infiltration. Moreover, the lesion MDM and microglia co-culture decreased the microglial-associated inflammatory cytokine expression, such as IL-1β, which further promoted lesion site healing and neurological functional recovery (Greenhalgh et al., 2018).

## The roles of lymphocytes—Friends or foes?

The adaptive immune system constructs a crucial defense barrier against pathogen invasion *via* a lymphocyte-mediated immune response. Under normal conditions of the CNS, sole adaptive immune components are present in the meningeal lymphatic system (including T, B, and dendritic cells), which provide immune surveillance and protection for neurons (Louveau et al., 2015). After an SCI, the BSCB damage leads to lymphocyte infiltration, and these peripheral immune cells are spontaneously transported into the lesion microenvironment. Although a previous study found that the overall inactivation of T and B cells promoted axon regeneration and neurological functional recovery post-SCI, recent studies suggest that T and B cell-mediated adaptive immune responses exert complex effects during the repair process (Wu et al., 2012).

### B cells

Mechanically, B cells exert the immune response in the CNS by mediating the antibody-associated complement activation and inflammatory cell recruitment. Pathophysiologically, the peripheral B cell populations underwent reduction owing to the surplus of glucocorticoids and norepinephrine induced by hypothalamic–pituitary–adrenal axis dysregulation post-SCI. However, due to the BSCB disruption-induced influx, the B cell elevation was detected in the spinal cord lesion site (Oropallo et al., 2014).

Giovanna et al. demonstrated that the glycoengineered anti-muCD20 antibody-mediated B cell blockade is conducive to SCI recovery. First, the inhibited pro-inflammatory factors and cytokine expression significantly decreased the neuron casualties and histological damage post-SCI, namely, NFκB, GFAP, and nitric oxide synthase. Moreover, the B cell inactivation modulated the immune response condition of other immune cells, such as T cells, NK cells, and microglial, which create the neuroprotective immune microenvironment in a lesion (Casili et al., 2016). Likewise, the B-cell knockout was demonstrated to exert neuroprotective effects against SCI, including improved locomotor recovery, decreased lesion volume, and immunoglobulin secretion. A possible explanation for neuroprotection is B cell inactivation-induced maturing of plasma cells and antibody deficiency (e.g., IgM, IgG) that are beneficial for SCI recovery (Jones, 2014).

### T cells

Soon after SCI, heterogeneity of T cell influx is involved in immune microenvironment modification, such as T-helper (Th) cells, including Th1, Th2, Th17, and Treg cells (Roy and Awasthi, 2019). Catarina et al. showed that Th1 cells are indispensable for the anti-inflammatory milieu reconstruction and tissue repair post-SCI. Th1 cell deletion not only disrupts the balance between anti- and pro-inflammatory responses in the spinal cord microenvironment but cumbers the functional recovery post-SCI (Raposo et al., 2014). In addition to the beneficial functions, Th1 cell activation leads to detrimental results. Under the chronic spinal cord compression condition, Th1-expressed cytokines, such as TNF-α and IL-6, induce the transformation and activation of the M1-type macrophages. As a consequence, the detrimental inflammatory cascade in the lesion site was exacerbated by the accumulation of M1 macrophages, which gradually resulted in extensive demyelination and neural tissue necrosis (Hirai et al., 2013). Compared with Th1 cells, which have a mixed contribution, Th2 cells are conductive to SCI recovery and neuroplasticity in the SCI repair process. The myelin basic protein activated T cells (MBP-T) transition is a crucial CNS trauma-induced autoimmunity regulation, which is regarded as a protective autoimmunity mechanism during SCI. Activated MBP-T cells can be transformed into either Th1 (immunity and phagocyte activation type) or Th2 (immunity and phagocyte inactivation type) subtype under certain circumstances. He et al. found that the Th2-dominant condition in the CNS facilitates neuron survival and axon remyelination post-SCI. Moreover, promoting the MBP-T transformation from the Th1 to Th2 subtype and raising the Th2 quantity is beneficial for SCI recovery (Hu et al., 2016).

Recent studies have proven that Treg cells act as an immune orchestrator in the hostile lesion microenvironment, which can suppress the overactive immune responses in the CNS. For instance, the Nrg-1 infusion post-SCI influences the T cell phenotype transformation, which exerts immunomodulatory roles. In the Nrg-1-administrated SCI model, the Treg cell population increased remarkably in both spinal cord lesion tissue and peripheral blood (Alizadeh et al., 2018). Liesz et al. found that in CNS ischemia models, the HDAC inhibition-induced Treg cell formation and activation not only ameliorated the neuroinflammation but also promoted neurological recovery. During this process, Treg cell-expressed IL-10 suppressed the M1 microglia transformation and leukocyte activation and inhibited the pro-inflammatory cascades in the brain ischemia lesion site (Liesz et al., 2013). Interestingly, the absence of Treg cells at the subacute or chronic stage inhibited the neurological recovery following SCI. In contrast, an earlier depletion of Treg cells before the injury was beneficial for SCI repair (Raposo et al., 2014). Consequently, the Treg cell-mediated neuroprotection is still subject to the constraints of time and space during the SCI pathological process.

## The immune depression post-SCI—The great recession

Cellular vitality and metabolic status are crucial for neurological recovery post-SCI. Unfortunately, during senescence, the regenerative and inflammatory response properties tend to reduce. A clinical cross-section study discovered that the immune function frailty (immunosenescence status) onset prematurely in patients with SCI. As a typical characteristic of immunosenescence, the T cells phenotype transformation from naive to memory was detected in younger patients with SCI. Under this circumstance, the secretion and proliferation capacity of T cells declined remarkably, which induced SCI-related immune deficiency (Pavlicek et al., 2017). Moreover, the cytokine expression and immune response activity of T and NK cells declined post-SCI (Schwartz and Raposo, 2014). SCI-induced immune depression syndrome (SCI-IDS) is another type of systemic maladaptive immune dysfunction post-SCI. Unlike immunosenescence, SCI-IDS is a neurogenic immune paralysis scenario. Owing to the SCI-induced immune system-associated dysautonomia, the immune response was found to be profoundly depressed. As a consequence, the SCI-IDS onset not only conjugated with leukopenia and leukocyte dysfunction but also facilitated the life-threatening infection spreading and tissue damage (Kopp et al., 2013). As the largest immune organ, the spleen is the first victim of the SCI-IDS condition, which is caused by sympathetic over-activation. Moreover, the SCI-caused spleen dysfunction deteriorated immunosuppression. For instance, the supraphysiological levels of norepinephrine in the splenic microenvironment post-SCI impaired the immune activity and cytokine secretion capacity of T cells, which accelerated the T cell exhaustion status (Zha et al., 2014). Of note, the splenic sympathectomy was shown to accomplish reversing the immune depression status post-SCI (Schwab et al., 2014).

## The immune organs and SCI recovery—The familiar strangers

**Figure 3:**
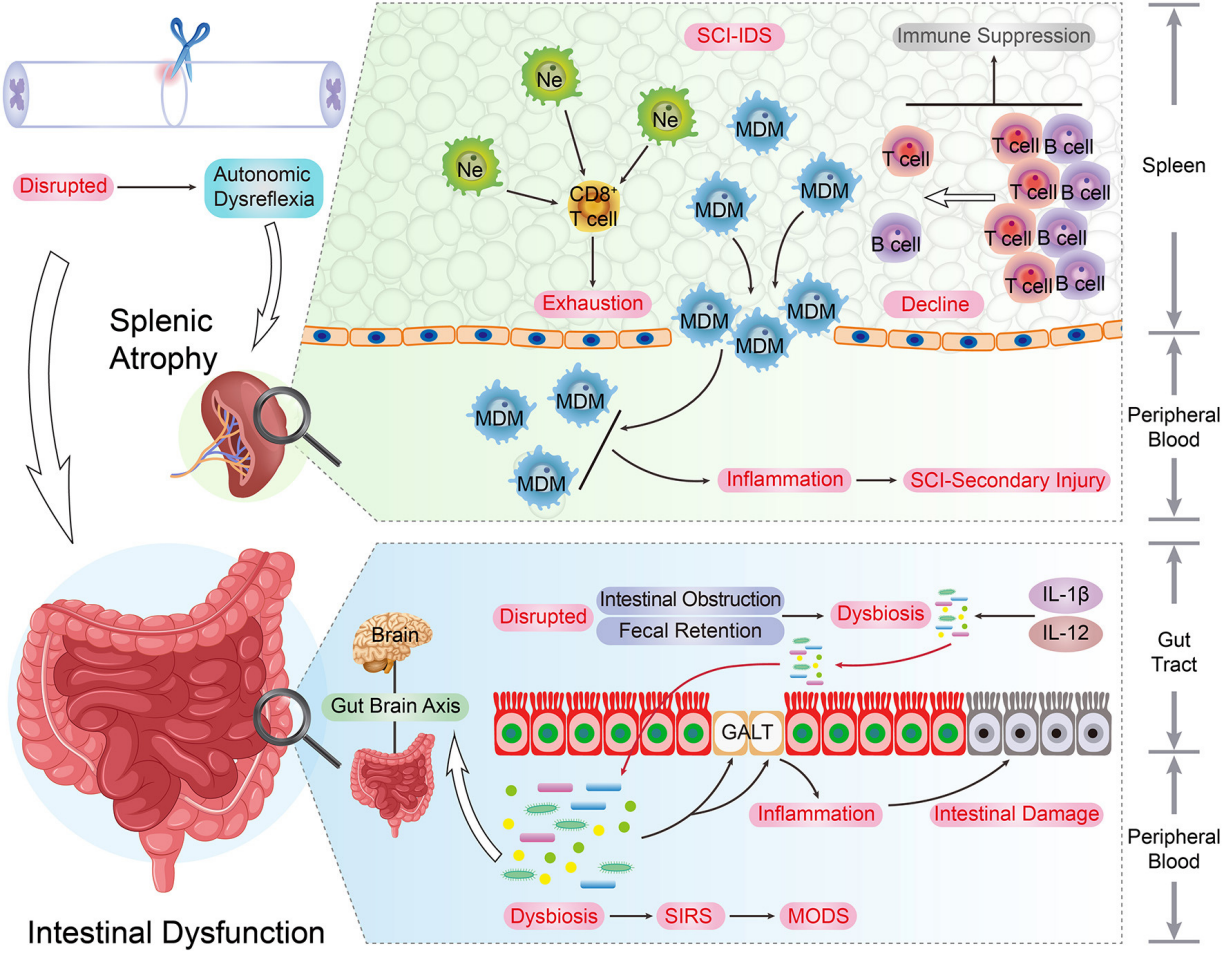
The immune organs and SCI recovery. As the development and storage places of immune cells, immune organs are indispensable for the immune system's smooth and effective operation. The upper spinal cord segment injury led to the autonomic dysreflexia status. Under this circumstance, the excessive norepinephrine and activated β-AR signaling result in adverse spleen dysfunction, such as splenic atrophy and immune cells secretion inhibition, and the quantity of peripheral and splenic CD4^+^ and CD8^+^ T cells underwent a decrease post-SCI. In addition, due to the surplus norepinephrine stimulation post-SCI, the immune activity and cytokine secretion capacity of splenic CD8^+^ T cells were impaired. These changes can be regarded as the typical hallmarks of SCI-IDS. Post SCI, the stress-induced intrinsic enteric nervous system interruption results in intestinal complications such as intestinal obstruction, fecal retention, and infections. As consequence, this situation raises the incidence of dysbiosis and induces immunity dysfunction, systemic Inflammatory response syndrome (SIRS), and multiple organ dysfunction syndromes (MODS). Moreover, the SCI-induced pro-inflammatory cytokines up-regulation (IL-1β and IL-12) takes place in the gut, and the intestinal immune factors disorder condition is parallel with beneficial bacteria reduction extent.

The spinal cord injury triggers systemic and extensive inflammatory and immune responses. Nevertheless, most studies have only paid attention to nerve regeneration and its regulation mechanisms due to the CNS centralized conception, and the rest of organs and tissues dysfunction has long been overlooked. As the development and storage places of immune cells, immune organs are indispensable for the effective operation of the immune system. Recent studies have focused on SCI-induced immune organ pathological modifications and immune function influences (Figure 3).

### The spleen and SCI

The spleen is the biggest lymphatic and immune organ. Physiologically, the sympathetic nervous system manipulates the immune defense function of the spleen, such as peripheral blood leukocyte filtration. The upper spinal cord segment injury disrupts the neural connection between the upper-level inhibitory signal and post-ganglionic circuits, which leads to autonomic dysreflexia status. Under this circumstance, the excessive norepinephrine and over-activated β-AR signaling resulted in adverse spleen dysfunction, such as splenic atrophy and immune cells secretion inhibition, and these changes were recognized as the typical hallmarks of SCI-IDS (Noble et al., 2018). This was illustrated by the decline of peripheral and splenic CD4^+^ and CD8^+^ T cell quantity after SCI (Zhang et al., 2013).

The spleen is the primary storage and refinery organ of MDMs. Despite the immune cell secretion inhibition, SCI triggers the release of MDMs into the peripheral circulation. These peripheral MDMs are further infiltrated into the SCI lesion and deteriorate the inflammatory response of the SCI secondary injury. Blomster et al. noted that the SCI-induced MDM infiltration and accumulation were strongly suppressed by splenectomy, promoting neurological recovery and alleviating the immunosuppression condition (Blomster et al., 2013).

Under physiological conditions, the splenic lymphocytes are tolerant to CNS endogenous antigens. However, immune tolerance can be compromised owing to SCI-induced BSCB disruption and CNS antigen exposure. When these endogenous molecules were captured by splenic lymphocytes, the autoimmune reactions were elicited, and the roles they played depended on the characteristics of target antigens. For example, by producing neurotrophic factors and antithrombin, myelin-reactive T cells enabled the protective autoreactive immune response post-SCI (Schwartz and Raposo, 2014). In contrast, due to the norepinephrine stimulation post-SCI, the immune activity and cytokine secretion capacity of splenic CD8^+^ T cells were impaired (Zha et al., 2014).

### The gut and SCI

The gut microbial system contains trillions of gastrointestinal resident microbes and was recently recognized as an independent organ for its self-reliant metabolism and absorption regulation functions (Fung et al., 2017). Moreover, as the key component of the gut–brain axis networks, gut microbiota exerts critical influences on CNS physiological or pathological modulation by means of neuroactive metabolites and neurotransmitters, for example, short-chain fatty acids, GABA, and dopamine. For instance, under the autoimmune encephalomyelitis condition, the transplantation of *Bacteroides fragilis* alleviates inflammation reaction and enhances the activity of the Treg cells (Jogia and Ruitenberg, 2020).

Post SCI, the stress-induced intrinsic enteric nervous system interruption may result in intestinal dysfunction, such as intestinal obstruction, fecal retention, and infections. Consequently, this situation raises the incidence of dysbiosis and induces holistic complications, such as immunity dysfunction, systemic inflammatory response syndrome, and multiple organ dysfunction syndromes. Remarkably, the SCI and dysbiosis form a vicious circle, and the dysbiosis triggers the mucosal immune cell activation in gut-associated lymphoid tissues (GALT), which eventually deteriorates the lesion inflammatory size and neurological impairment (Kigerl et al., 2016). In patients with traumatic SCI, although the gut microbiota diversity has reduced tremendously, bacterial abundance augment has been detected, such as the proteobacteria, bacteroides, and verrucomicrobia phyla. Interestingly, these bacterial spectrum modifications are related to the extent of neurological dysfunction of SCI (Zhang et al., 2018). Moreover, the SCI-induced pro-inflammatory cytokine upregulation (IL-1β and IL-12) takes place in the gut, and the intestinal immune factors disorder condition is parallel with the extent of reduction of beneficial bacteria (Gungor et al., 2016).

## Therapeutic perspective—A silver lining

The spinal cord injury-induced catastrophic neurological disability is currently incurable. Recent studies demonstrated that comprehensive decoding and manipulation of SCI-induced immune microenvironment modification are crucial to the spinal cord's neurological recovery. Essentially, these breakthroughs propose promising immune-associated candidates for further SCI studies and clinical treatment. Generally, based on the immune checkpoints and pathological mechanisms of SCI, the immunotherapy principles should be targetable, for instance, the inflammation regulation-oriented (innate glia or WBCs) and immunomodulation-oriented (adaptive immune cells or organs) treatments.

Minocycline, as a semi-synthetic second-generation tetracycline, has been used as an antibiotic for decades. Recently, the neuroprotective characteristics of minocycline were extensively explored, which are distinct from its well-known antibiotic identity. More specifically, by inhibiting the canonical inflammatory pathway, minocycline prevents microglia activation, and thus, the secretion of inflammatory mediators and cytokines was found to be restricted (Bowes and Yip, 2014). In a randomized clinical study involving 52 patients with SCI, the neurological treatment effect and safety of minocycline were affirmed (Casha et al., 2012). Remarkably, the minocycline SCI treatment dosage was optimized to 90 mg/kg on the first-day post-injury, followed by half dosage per day (Chio et al., 2021).

At present, the roles of immune checkpoints in CNS traumatic injury-related neuroimmune and neuroinflammatory responses are finely discovered. The programmed cell death protein 1 (PD-1) is a crucial T cell-related immune checkpoint for anti-tumor immunotherapy. Recent studies found that PD-1 can be expressed by multiple SCI inflammation participants, such as Treg cells and glial cells. For instance, the M1/M2 phenotypes transformation in microglia post-SCI was closely related to PD-1 signaling activation. The PD-1 upregulation promotes the anti-inflammatory cytokine expression and drives the microglia M2 polarization. Meanwhile, the PD-1 signaling suppresses the M1 phenotype microglia formation. As a consequence, the PD-1-induced M2 microglia polarization inhibits the neuroinflammation ignition post-SCI, which is beneficial to SCI function recovery (Zhao et al., 2021); PMID: 33877518). Likewise, the programmed death-ligand 1 (PD-L1) is a vital inhibitory immune checkpoint, which was shown to regulate the neuroimmune responses in the CNS. The *de novo* expression of PD-L1-induced positive reactive astrocyte enrichment occurred around the lesion site of the CNS traumatic injury. With the accumulation of these reactive astrocytes, the inflammatory cell recruitment and activation were repressed, which retarded the tissue damage and neurological disability. In contrast, the PD-L1 signaling inactivation promoted inflammatory cell infiltration and deteriorated the functional outcomes of traumatic brain injury (Gao et al., 2022).

The stimulator of the interferon gene (STING) is a critical immune sensor that recognizes the foreign DNA for anti-tumor immunity (Hu et al., 2022). By regulating inflammatory factor expression, such as interferon I (IFN-I), the STING signaling pathway promotes the clearance of neoplastic and pathogens (Donnelly et al., 2021). Besides, STING facilitates the neuroimmune and inflammatory response in CNS pathophysiology disorders, which provides a novel potential strategy for SCI treatment. In an SCI lesion, STING upregulation can be detected, which is parallel with the inflammatory cytokine expression in the lesion. Wang et al. found that STING can be captured and phosphorylated by TANK-binding kinase 1 (TBK1). Thus, the downstream inflammatory pathway targets of STING, such as NF-kB and MAPK, were activated to trigger the inflammation progress (Wang et al., 2019). Instead, the STING ablation relieved the spinal cord inflammation and promoted SCI recovery via the TBK1/STING pathway.

Human immunoglobulin G (hIgG) is an effective FDA-approved immunomodulator that is widely applied in clinical CNS immunotherapy. A recent study indicated that the hIgG treatment conferred neuroprotective and immunomodulatory activities in both a dose- and time-dependent manner. Notably, studies optimized the effective dosage of hIgG (2 g/kg) to execute the immunomodulatory effects, such as inflammatory infiltration attenuation, immune cell inactivation, and vascular integrity improvement (Chio et al., 2019).

Notwithstanding these promising immunotherapy breakthroughs, owing to the BSCB isolation, effective drug penetration and spinal targeting approach remain to be adequately explored. With developments in nanodrug loading technologies, effective immunotherapy drugs can be delivered to the lesion site, which would endow SCI clinical treatment with favorable weapons (e.g., the novel synthesized lesion targeting nanoparticles named triblock copolymer-MMPs targeting peptide [PPP-ACPP]). In a study, the etanercept (a well-established TNF-α antibody, ET) was released and accumulated into the lesion site *via* the ET- PPP-ACPP loading manner. Thus, the SCI epicenter ET enrichment not only decreased the M1-type macrophage quantity but also alleviated the secondary injury-associated inflammation cascade (Shen et al., 2021).

Recently, a novel subset of neutrophils (the N2 cells) with neuroprotective capacities was identified, providing a promising candidate for neurorestorative immunotherapies. Unlike the traditional neutrophils, these novel N2 neutrophils and CD14^+^ cells are unable to produce pro-inflammatory cytokines and chemokines. Conversely, N2 cells induce a robust axon regeneration post-SCI *via* a dependent manner, such as NGF and IGF1 (Sas et al., 2020).

We know that SCI-induced intestinal dysfunction deteriorates immune disorders. Unfortunately, SCI-caused sensory deficiency makes these gut complications more undetectable, which aggravates gut microbiota dysfunction and dysbiosis. Consequently, timely gut management is essential for gut barrier strengthening and complication reduction post-SCI. A recent study reported that an earlier oral administration of a probiotic cocktail (named VSL#3, eight probiotic strains enriched mixture) not only benefits the gut lactic acid–producing bacteria abundance elevation but also promotes neurological recovery (Kigerl et al., 2016). Another study in rodents indicates that timely oral feeding of diluted fecal matter from healthy counterparts may rescue SCI-induced dysbiosis, intestinal permeability disorder, and neurological disability *via* a short-chain fatty acids anti-inflammation manner (Jing et al., 2021). Consequently, compared with sophisticated surgery operations and complicated immunotherapy, these gut-associated breakthroughs provide promising SCI treatment that is more feasible for patient daily care scenarios, for example, the oral administration agents of gut microbiota.

Credible biomarkers to predict clinical outcomes are crucial and desirable. Unfortunately, owing to the complicated pathological changes of SCI, establishing a reliable prognosis system is a challenging task. As the early infiltrated immune cells, the neutrophils are essential for the immunomodulation and inflammation reaction of SCI. Recently, a retrospective study involving 163 patients found that acute neutrophilia (circulating neutrophil > 7.7–8.0 × 10^9^ cells/L) post-SCI was positively correlated with the severity of neurological impairment. Moreover, the ratios of peripheral blood neutrophils and lymphocytes were demonstrated to be closely related to patient respiratory system infection (Jogia et al., 2021).

## Conclusion

**Figure 4:**
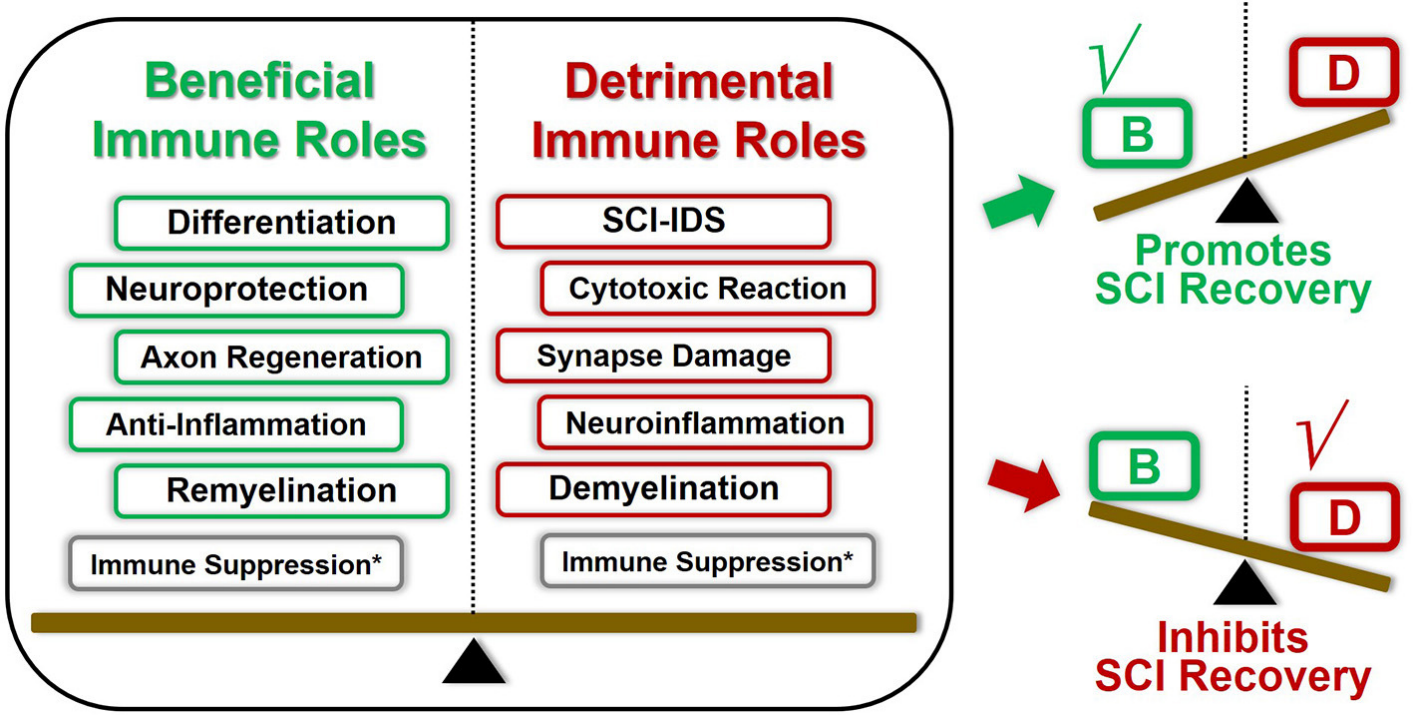
Double-edged immune factors modulate the overall immune landscape SCI recovery. Most of these aforementioned immune factors played both beneficial and harmful roles in regulating SCI pathological repair process. The overall outcomes of SCI pathological repair are the superimposed results of multiple immune elements. For instance, once the beneficial immune roles overcome these detrimental counterparts under certain conditions, the overall immunomodulation tent to promote SCI repair, and vice versa. Consequently, coordinating the panoramic immune landscape of SCI pathophysiological changes will provide us deepening understanding of how immune factors modulate the SCI recovery process. *The immunosuppression effects could be both beneficial and detrimental toward SCI recovery, which presents various characteristics due to the spatiotemporal difference.

Traditionally, owing to the BSCB seemingly creating an “immune privileged” area, immune elements tend to be treated as “hidden players” in SCI, which garnered less attention in previous studies. However, recent studies present the panoramic immune landscape of SCI pathophysiological changes, providing a deep understanding of how immune components modulate the SCI recovery process (Figure 4). Around the spinal cord epicenter, the SCI-associated immune response can be immediately aroused in either an innate or adaptive manner.

These activated immune cell-produced immune molecules and antibodies modulated the immune organ's function and even induced immunosuppression conditions. Remarkably, the aforementioned neuroimmune regulation mechanisms are likely to play both beneficial and harmful roles in governing the SCI pathological repair process (Table 2). First, the same categories of immune cells tend to present various characteristics owing to the spatiotemporal difference. In addition, the overall outcomes of SCI pathological repair are the superimposed results of multiple aspects of immunomodulation. Consequently, favorable orchestration of these pros and cons aspects of neuroimmune factors is prone to turn the injured spinal cord into a regenerative competent state, eventually promoting SCI recovery. Even so, specific immune mechanisms underlying SCI remain to be illustrated.

**Table 2:** The effects of SCI immunomodulation-associated cytokines.

**Cytokines**	**Producers**	**Main effects and regulation**	**Roles in CNS recovery**
IL-1β	Astrocytes MDM	CD4+ T cells migration and polariztion& Neuroinflammation progression (Liu et al., 2019) Tissue inflammation damage (Greenhalgh et al., 2018)	Negative Negative Negative
IL-6	Astrocytes Microglia Th1 cells	Neuroinflammation progression (Liu et al., 2019) Promotes A2 phenotype Astrocytes formation (Joshi et al., 2019) Promotes M1 phenotype Microglia formation (Hirai et al., 2013)	Negative Positive Negative
IL-10	Astrocytes Treg cells	CD4+ T cells migration and polariztion& Promotes neurological recovery and inflammation limitation (Liu et al., 2019) Treg neuroprotection effect, inhibits M1 formation (Liesz et al., 2013)	Negative Positive Positive
IL-15	Astrocytes	Promotes CD8+T and NK cells induced immune related damage (Li et al., 2017)	Negative
IFN-γ	Oligodendrocytes	Promotes CD8+T cells induced OPC damage (Kirby et al., 2019)	Negative
TNF-α	Microglia Th1 cells	Promotes A2 phenotype Astrocytes formation (Joshi et al., 2019) Promotes M1 phenotype Microglia formation (Raposo et al., 2014)	Positive Negative
MMPs	MDM Neutrophils Exotic	Promotes ECM formation and lesion recovery (Hong et al., 2017) Promotes neurological recovery (Lee et al., 2011) Inhibits M1 phenotype MDM formation and secondary cascade (Shen et al., 2021)	Positive Positive Positive
MCP-1	Astrocytes	CD4+ T cells migration and polarization, proinflammation (Liu et al., 2019)	Negative
Drp-1	Microglia	Promotes A2 phenotype astrocytes formation and lesion damage (Joshi et al., 2019)	Negative

Moreover, some technical challenges are expected to be solved, for instance, the stable and safe loading-delivery system and the specific cellular or organ of targeting technique. Based on these favorable breakthroughs, it is reasonable to believe that these uncertainties will be solved gradually by deepening the understanding of SCI-associated neuroimmune mechanisms. Altogether, further exploring immune-based mechanisms and treatment targets will broaden SCI clinical therapy perspectives.

## Author contributions

LZ-G: drafting figure and writing section–The immune roles of spinal cord glial cells–Far beyond the glia. YF: writing sections–The immune roles of peripheral immune cells–The unfavorable guys? and The roles of lymphocytes–Friends or foes?. CP: writing section–The immune depression post post-SCI–The great recession and revising the manuscript. ZB-Y: conceptualization, writing sections–The immune organs and SCI recovery–The familiar strangers, Therapeutic perspective–A silver lining, and conclusion and revising the manuscript. All authors contributed to the article and approved the submitted version.
